# Our Contemporaries

**Published:** 1912-06

**Authors:** 


### OUR CONTEMPORARIES

THE *Buffalo Commercial* has recently published an interesting article describing how the city refuse is disposed of. Waste paper alone amounts to 22 tons a day, worth $10.00 per ton. Broken glass is re-melted. About 2,000 unbroken bottles and 300 milk bottles are saved daily. The last are sold to the original owners at one cent each. It is some comfort to know that the bottles are sterilized but, for Heaven's sake doctor, smash your urine bottles before throwing them out.

PRACTICAL *Medicine* of Delhi, Ind., advertises "an ointment which is free from all poisonous substances and is claimed to be very successful in the treatment of all kinds of skin diseases." It is manufactured at Sukkur—and probably sold to Sukkurs.

THE *Homeopathic Envoy* comments as follows on Col. Crego's article:

MEDICAL SCIENCE VS. HOMEOPATHY.—Here is an illustration of why the latter, even in the hands of one not very learned, is better than the other, that is, better for the patient. In a paper on "Meningitis" Dr. F. S. Crego (*Buffalo Medical Journal,* March) writes: "Pachymeningitis externa is a slow inflammation of the external layer of the dura. As you remember" (which probably you do not) "the dura is the periosteum of the internal table of the skull as well as the external covering of the brain. This disease is rather common, and is so frequently mistaken for the other disease that it is well worth while to give it a few moments of consideration." Here follows a very excellent dissertation on the various causes of this ailment. Now, the point is this: That to acquire this exquisite and enviable skill in diagnosis must take years of hard and patient study. Afterwards when a case is presented and the diagnosis of pachymeningitis made, the question arises, what is to be done? The learned man at this point looks up in a dazed manner and too often in effect replies, "I do not know! I merely study the manifestations of the disease," or else he gives some routine treatment. A good homeopath might never have heard of pachymeningitis externa, yet from the symptoms presented by the patient, he might be able to do what the learned one could not, namely, cure the case.

We were going to say something scurrilous but, after all, do we as a general proposition have to stagger and fall like one with locomotor ataxia, in our treatment, just because we are in the dark as to diagnosis? Really we relieve quite a good many cases but we do not thoroughly understand any.

---

We beg leave to call the attention of the *Journal of the A. M. A.* to page 24 of the advertising pages of *Munsey's Magazine* for May, next to frontispiece. Here will be found an advertisement of a medicinal preparation advertised on page 19 of the advertising pages of the *Journal of the A.M.A.* There is an old proverb that people should not let their children throw stones at green houses till their own windows are boarded up for the summer vacation.

